# Extra Supplement.—The Nursing Mirror

**Published:** 1893-10-21

**Authors:** 


"The Hospital" Nursing Mirror.

Being the Extra Nursing Supplement of "The Hospital" Newspaper.

[Contributions for this Supplement should be addressed to the Editor, The Hospital, 428, Strand, London, W.C., and should have the word "Nursing" plainly written in left-hand top corner of the envelope.]

### News from the Nursing World.

### OBSERVANT VISITORS.

A series of cordial and enthusiastic receptions marked the visit to Scotland of the Royal bride and bridegroom. To nurses, the chief interest of the whole proceedings centres in the wards passed through by the Duke and Duchess of York, and particularly in the kindly interest taken in individual patients. The Prince of Wales has always been remarkable for his fine tact and comprehension of details appertaining to institutional management, and it is pleasant to see that his son inherits both characteristics. The Duke and Duchess expressed warm approval of the arrangements made for the comforts of the occupants of the new Nurses' Home at Edinburgh, which they inspected very thoroughly.

### OUR CHRISTMAS COMPETITIONS.

We anticipate that an immense variety of garments will this year be made by our readers for the adult sick poor who have to spend their Christmas Day within hospital walls. The parcels which are the result of our annual competition receive such a hearty welcome at each institution to which they are sent, that we hope to see them increase in bulk and in number every year. The prizes will be: (1) For the best pair of socks knitted by a nurse, 5s.; (2) for the best pair knitted by any Hospital reader, 5s.; (3) for the best made flannel shirt, 10s.; (4) for the best flannel or flannelette bed-jacket, 10s.; (5) for the best flannel petticoat, 10s.; (6) for the best made and simplest shaped dressing gown made and cut out by a nurse, 20s. Long seams may be done by machine. With the view of enabling an estimate to be formed of the progress made, we shall be glad to receive the names of competitors, and to hear which of the six prizes each will enter for. All parcels must reach The Hospital Office, 428, Strand, in the first week of December, and they should bear the words "Needlework Competition" in left-hand corner of address label, and also the name of the sender.

### THINGS THAT ARE WANTED.

The record of a district nurse's work is often put down merely as so many visits paid annually to a certain number of patients. But that gives a most inadequate idea of what she has really accomplished. Each patient being probably one of a different family circle, the nurse finds a new cluster of interests surrounding every individual case. The poor are necessarily close together in their home life, and a visit to one sick person means contact with many other people in the course of a day. All this increases the strain on a nurse's sympathies and resources, and, moreover, all the patients have many needs, with some of which the district nurse can cope, whilst others are beyond her powers. Whatever the stock from which she is free to draw, it is nearly always insufficient to meet the demands upon it; therefore, all who sympathise with the grand work of nursing the sick in their own homes might prove their practical interest by sending any suitable appliances to institutions which undertake this labour. Nothing can come amiss for those who have so few comforts of their own, and the list of their requirements embraces everything, from a water bed to an ear trumpet.

### NURSES FOR THE INSANE.

Nursing in provincial asylums has made great progress of late years, but much still remains to be done as regards the systematic training of intelligent women to take charge of mental cases. The authorities at Berrywood, Northampton, have set a good example by arranging to receive paying pupils to whom every advantage of theoretical and practical teaching is given. There is always a demand for qualified nurses for private cases of mental disease. Such patients, whether they be slightly or seriously affected, always need skilful attendants, and the latter are, therefore, sure of constant and well-remunerated employment. It is very remarkable, therefore, that so few women will be at the pains to educate themselves for this responsible and well-paid work. The pupils at Berrywood Asylum will receive a certificate on the satisfactory completion of a year's training. There are vacancies for not less than three candidates just now, and nurses who have finished their course of general hospital training would do well to consider the advantages held out at Berrywood. All particulars of fees, &c., can be learnt by writing to the Medical Superintendent at Berrywood Asylum.

### GOOD ENOUGH FOR THE POOR!

Perhaps this is a doctrine more often acted upon than put into words. Good men and women feel no shame in giving away only those things that they have "quite done with." It is reasonable enough to hand on what is no longer needed by ourselves, but it is well to make sure that it is worth anyone's receiving. Even the destitute who are willing to accept the rich man's crumbs, discriminate between gifts of old, carefully mended garments which make even poverty respectable, and ragged cast-off clothes from less thoughtful donors. But it is not only in the clothing of their bodies that the poor are apt to be treated slightingly. There is the more serious evil of giving them so-called nurses who are considered "good enough for the poor," although those who say so would never trust their own dear ones in such ignorant hands for half an hour. Want of knowledge which simulates helplessness should be the strongest possible claim for the best kind of help. If an intelligent and prosperous person needs in sickness a specially qualified attendant, how much more so the poor bread winner without knowledge of the common laws of health. His chances of life being for this reason, amongst others, considerably less than those of the well-to-do patient, he should have a nurse of greater, certainly not of less, training and experience. A homely, ignorant woman may strike the cottager as

"a nice friendly body," but no confidence is placed in her judgment, though she is probably willing enough to humour the superstitious dislike to fresh air and water, and also to foster the propensity to indulge in drugs not ordered by the doctor. In nursing as well as nourishment only the best and safest kinds are "good enough for the poor!"

### A LASTING MEMORIAL.

A memorial to the late Nurse Groser has been erected in Benwell churchyard, where she was laid to rest last April. The monument is of Peterhead granite, in the form of a rustic cross, and has been raised at the express wish of, and by subscriptions from, those to whom Nurse Groser in her capacity of parish nurse devoted herself for nine years. A large number of people were present in the churchyard. The Rev. Canon Bromley remarked on the fact that Dr. Hodgkin, by whom the stone was unveiled, had been largely instrumental in securing the services of the late Nurse Groser to the sick poor of the district, in which she became greatly beloved and universally valued.

### LOST OPPORTUNITIES.

It would be very difficult to secure accurate figures as to the number of children who go to Margate every year weak and delicate, and return to their homes strong and sound. Disease is arrested and feebleness is soon banished in that wonderful life-giving air. The visitors are of all classes—certainly many of them are well-to-do—yet these, having secured to their dear little ones the blessing of restored health, turn their backs on this watering-place, and hasten to luxurious homes, with no thought for those equally in need of sea breezes, but unable to afford the expensive food and costly lodgings. Surely each person who leaves Margate in renewed health should leave a donation to the Sea-Bathing Infirmary, and thus assist to reopen the closed wards. Of all the sad sights in the world that of the empty wards at Margate is one of the worst. Beautiful rooms, and every facility for the cure of scrofula, yet all this is wasted, and patients needing treatment cannot have it because funds are not forthcoming to support one of the most valuable of English institutions.

### INDIA.

The Sanitary Commissioner's report of the condition of the Punjaub during the past year has considerably alarmed the residents therein; and a yet more serious account is given of the N.W. provinces and Oudh, the total mortality in the latter places being 1,168,077. Two hundred thousand deaths were due to cholera. The victims to small-pox were 8,000, which is only a third of the number sometimes registered. The Hospital correspondence on nursing uniforms has excited much interest in India, where it is compulsory in the "nursing service," and also for Lady Roberts' staff, to wear outdoor uniform. The nursing sisters not unrequently long for this to be optional, so that they might sometimes discard their pretty but very small bonnets for head-gear more suited to the tropics.

### A PLEASANT ENTERTAINMENT.

A reception was held in the Administration Building of the New York Hospital Training School for Nurses, on Saturday evening, September 30th, by the graduating class of eleven members. The parlours were beautifully decorated with plants and flowers, and the nurses in their blue and white costumes, with large white kerchiefs, caps, and cuffs, received their invited guests. The exercises were very short, consisting only of the presentation of diplomas and gold badges by one of the governors of the hospital, and then the supper-room was thrown open and refreshments were served. The tables were artistically arranged, and the lovely flowers which adorned them were gifts received during the day by the members of the graduating class. Some excellent music gave much pleasure to the guests, and the proceedings concluded with "Auld Lang Syne."

### CHICAGO AND IRONWOOD.

Since the early summer the healthy condition of Chicago has been most marked, and many nurses actually migrated from the city to smaller towns in search of sick people. Five members of the Illinois Training School spent the summer at Ironwood, Northern Michigan, where an epidemic of typhoid prevailed, and their valuable services were greatly appreciated and welcomed. Ironwood is considered a remarkably healthy place, with peculiarly clear and bracing air, but a contaminated water supply this year brought about a visitation of typhoid. The Armoury was made into a temporary hospital, and the patients were all taken there and cared for at the City's expense. The death-rate was commendably low, and the origin of the mischief was discovered and remedied. An unfortunate cat had wandered into the city water pipes and died there, and the water had doubtless been contaminated for some time previous to the outbreak of illness.

### SHORT ITEMS.

The October number of *Misericordia*, the monthly paper of the Guild of St. Barnabas, contains an excellent summary of the position and aims of the Royal National Pension Fund for Nurses.—An excellent programme of winter work has been prepared by the London Young Women's Christian Association. The members have the opportunity of attending educational and civil service classes, gymnastic exercises, and lectures on hygiene, sanitation, nursing, and cookery. Employment agencies are also associated with the L.Y.W.C.A., 16a, Old Cavendish Street.—Mr. Andrew Clark, F.R.C.S., is giving a course of lectures on First Aid and Sick Nursing, at the Polytechnic, in connection with the St. John Ambulance Association.—The Samaritan Hospital, Sioux City, Iowa, intends to organise a training school for nurses.—Mrs. Thomas, who received the Radford medal for proficiency in midwifery at St. Mary's Hospital, Manchester, in 1890, has just gained another medal, which was offered by the Medical Board of the same hospital for advanced knowledge in obstetrics, theoretical and practical, and could only be competed for by those already holding a midwifery diploma. Mrs. Thomas was complimented and congratulated publicly on the excellent paper she had sent in.—A conference of lady guardians was recently held at 83, Lancaster Gate, and the Countess of Meath read a paper on the subject of supplying trained nurses for the aged and infirm in the sick wards of workhouses. A committee was formed to consider the matter.

### On the Nursing of Diseases of the Nervous System.

### III.—APOPLEXY AND APHASIA, CEREBRAL TUMOUR AND ABSCESS.

*Diagnosis of Apoplexy.*—In some cases it may be difficult to say whether a person is suffering from the effects of alcohol or from mischief in the cerebral vessels. The fact of the breath smelling of alcohol should not negative the possibility of cerebral hæmorrhage. The pupils are generally equal and re-act to light in alcoholic poisoning. If there is evidence of hemiplegia, or if other symptoms are present as stertor, convulsions, rigidity of limbs, the diagnosis of cerebral disease is confirmed. Time will tell also, as a patient will sleep off the effects of drunkenness, hence it is well, if there is any doubt about a case, to treat it as if it were the more serious, put the patient to bed and watch him carefully.

Secondly, the coma which succeeds an epileptic fit may be mistaken for apoplexy. If a history of epileptic fits can be obtained, of course there may not be much difficulty.

Thirdly, injuries to the head may give rise to symptoms very similar to those of apoplexy. Quiet and rest are essential here also until further help is obtained.

Fourthly, narcotic poisoning may resemble apoplexy, especially if the latter is caused by hæmorrhage into the pons, for in such cases the pupils are contracted as they are in opium poisoning. Treatment for narcotic poisoning is the very opposite of that for apoplexy, for the object is to rouse the patient, giving him hot coffee, walking him about, and using every means to keep him awake.

Fifthly, the effects of kidney disease may give rise to a set of symptoms similar to apoplexy. In such cases a purgative will be given, and the skin got to act, either by hot-air baths or by pilocarpin grain ($\frac{1}{8}$—$\frac{1}{2}$), given hypodermically, to try and get rid of the waste and poisonous material which the kidneys have failed to eliminate.

Coma may occur in diabetes also, and always ends fatally in such cases.

Sixthly, organic disease of the brain as tumour and meningitis causes coma. These diseases will be considered later on.

Lastly, high fever gives rise to a comatose condition. When there is hæmorrhage into the pons it has already been mentioned that the temperature is high, in other cases of cerebral hæmorrhage it is generally depressed.

*Aphasia.*—Complete hemiplegia on the right side of the body is accompanied by loss of speech, that is to say, when there is damage on the left side of the brain. We usually make use of only the left side of the brain for speaking, and this goes along with the greater use of the right hand; for in left-handed people it is paralysis of the left side, not of the right side which is accompanied by loss of speech; that is, they use mostly the right side of the brain for writing and for speech.

It will be easier to understand how speech is affected in disease if we consider what takes place when a child learns to speak. First he hears words; the ear must be able to receive the impressions; then there is a part in the brain, the centre for hearing, where the meaning of words is perceived; this region is connected with another centre, the speech centre in the brain which sends out motor impulses to another part of the brain from which the nerves of the tongue, lips, and larynx take origin. Thus a child who has never heard cannot speak. Later on a child learns to understand language by seeing words written as well as hearing them spoken, and can express them by writing as well as speaking, so that the eye and hand are connected with the speech centre.

In hemiplegia it is the speech centre which is affected; the hearing centre is all right, so a hemiplegic patient can understand what is said to him, but he cannot articulate, except a few words which he says automatically. This form of aphasia is called motor aphasia. There is another form, in which the hearing centre is affected, so that words are not understood. The patient speaks in this case, but he does not understand what he says, so he talks rubbish.

Again, in some cases both the centres for hearing and speech may be unaffected, but the nerves going to the tongue and lips paralysed; then the patient cannot speak, but if the arm is not paralysed he can then write. If one side of the brain concerned in speech be diseased, the other side may after a time take on some of the functions, and speech may be in some measure restored. This is always the case in children, in whom the brain is not fully grown.

Of course, no one when nursing an aphasic patient will forget that probably he understands all that is said, and the nurse will take especial care to find out his wants, and minimise in every way, as far as possible, the trial it must be, not to be able to communicate with those about him.

*Tumours of the Brain.*—There are a group of symptoms which occur in tumour of the brain independent of the situation of the tumour. These are headache, vomiting, and optic neuritis. The headache is usually severe and constant, persisting through the night. Vomiting is not preceded by nausea, but is often accompanied by attacks of pain. Optic neuritis is a change observed by means of the ophthalmoscope in the optic nerve at its entrance into the eye. The inflammation is very likely to go on to destruction of the optic nerve, and this causes blindness; but in the early stages optic neuritis may not interfere at all with sight.

Besides these symptoms there are others due to increasing interference with the functions of the brain, and these symptoms are dependent upon the particular position of the tumour. If the tumour is situated at the base of the brain, it may press upon the cranial nerves, destroying their function. For example, some of the nerves going to the muscles of the eyeball may be paralysed, and there will be squinting; or again, the auditory nerve may be interfered with, causing noises in the ear or deafness. If the tumour is situated in the cerebellum there are the usual symptoms of tumour, vomiting, headache, and optic neuritis, and there is giddiness, with tendency to fall when walking, so that the gait of a person with cerebellar tumour is very similar to that of a person drunk. There is also general muscular weakness, and sometimes spasm of the muscles of the neck. A tumour may be situated in some part of the motor tract, causing paralysis, or, if seated in the cortex, possibly spasm of certain groups of muscles (Jacksonian Epilepsy). A nurse by noticing exactly where these spasms begin and how they proceed may render very good service in enabling the doctor to diagnose the position of the tumour. The most common kinds of tumours are tubercular, syphilitic, or sarcomatous.

Besides general treatment, the question of operation arises, but this we shall not discuss here. Cases of cerebral tumour may go on for a long time, and they may even improve up to a certain point. It is often very difficult to know what to do with these cases, for they cannot be kept in hospital the whole time, yet they cannot earn their living, and besides having to be kept by their relatives, they need a certain amount of looking after, especially so as their eyesight goes. Without doing actual nursing, a nurse who is working in a district and can look after any such cases, trying to find any handiwork which is suitable, and cheering a very trying existence, would be doing a good work. Of course, towards the end of the case, when there may be more paralysis and loss of consciousness, much nursing is needed.

*Cerebral Abscess.*—The symptoms of cerebral abscess are similar in some respects to those of cerebral tumour, that is to say, there is headache, vomiting, and sometimes optic neuritis. There is often mental dulness, and there are symptoms depending upon the position of the abscess in the brain.

The first point of difference to be noted between cerebral abscess and tumour is that symptoms may develop very rapidly in the case of abscess, the illness having an acute onset. The abscess has in such cases probably been present for some time without causing symptoms. In some cases of cerebral abscess the temperature is raised, and there are rigors, but in other cases the temperature is never raised but is even subnormal. Another distinction between tumour and abscess is that a cause for the abscess can generally be found. The most common cause is ear disease; other causes are diseases of the nose, injury to the head, blood poisoning, and sometimes diseases of the lungs.

The treatment is surgical, and rests on the possibility of diagnosing the position of the abscess.

### Our Examinations.

Some very good answers have been given to our examination question for this month. Most of them, we are glad to see, put the immediate necessity for summoning the doctor in the first place, and then say what should be done pending his arrival. A few papers contain injudicious suggestions as to drugs. No unqualified person should dream of prescribing, but these answers show that there are people with a strong inclination to usurp the doctor's province and "suggest treatment." Ergot, iced champagne, blisters, cupping, inhalations, are amongst, but by no means all, the things proposed by some of our correspondents. Others have sent good and simple answers to our very plain question, showing that they are practical nurses, with an excellent knowledge of the best way to keep a patient quiet and tranquil, and not on any account to leave him alone until the doctor's arrival. Ice being seldom within reach in a cottage emergency, we are glad to find that cloths wrung out of the coldest water obtainable are referred to as useful outward applications.

October Question.

What is hæmoptysis? What should be done when it occurs (1) in a hospital, (2) in a cottage?

*Prize Answer.*

Hæmoptysis is spitting of blood from the lungs. It occurs in consumption, and sometimes in cases of heart disease and congestion of the lungs; in hæmoptysis the blood is coughed up; it is bright red, frothy, and often mixed with phlegm. The quantity varies—in slight cases only a few ounces are brought up, while in severe cases a pint or more may be expectorated. If a case occurs in a hospital the house-surgeon ought at once to be informed. The patient must be put to bed in a sitting posture, kept perfectly quiet, and not allowed to speak or excite himself. The ward must be kept cool, ice-bags applied to the chest, and pieces of ice given to suck.

When a case occurs in a cottage the patient must be got to bed in as airy a room as possible, without a fire. The doctor must be sent for, and in the meantime ice may be given if available. No stimulants must be allowed, and nourishment must be administered in a cold liquid form; it is most essential that the patient be quiet and anxious friends should be prevented from alarming him as to his symptoms.

M. Fry.

Examination Question for November.

If a case of cholera occurs in a cottage or in workmen's dwellings, what precautions should be taken by the nurse summoned to the case (*a*) as regards the patient, (*b*) the rest of the household, (*c*) and herself?

Answers must be sent in by November 4th. They must be clear and concise, written on one side of the paper only, accompanied by writer's name and address, and directed to "Nursing," care of Editor of The Hospital.

### The Nightingale Probationers.

ANNUAL VISIT TO CLAYDON HOUSE.

For sixteen years the "Claydon Day" has been a memorable event to the Nightingale probationers of St. Thomas's Hospital who are annually entertained by the Right Hon. Sir Harry Verney, Bart., President of the Council of the Nightingale Fund Training School for Nurses, and brother-in-law of Miss Florence Nightingale.

On Thursday, September 21st, twenty-eight probationers, accompanied as usual by Miss Crossland, the Home Sister, reached Claydon House, Buckinghamshire, at eleven o'clock and were cordially received and refreshed with tea, coffee, &c. At half-past eleven a short service was held in the lovely little church in the grounds and a helpful and practical address was given by the Rev. Llewellyn Kenyon Stow who referred to the unfortunate illness of the Rector which prevented his being present on the occasion. Towards the conclusion of his interesting sermon Mr. Ll. Kenyon Stow said:—

"A word to the women before me—a word to all women who work. A missionary returning home after some years absence was asked if he noticed any changes in England. 'Yes,' he replied, 'the women have woken up.' He was right. I need not warn you against that fussy activity which scamps real work, and cuts against a rich and human influence, turning women, as it does men, into mere clattering machines. I speak to you who have gifts, some, perhaps, great gifts, like those of St. Peter. But because you are women, those very gifts need special care. You throw yourselves more eagerly into work than men; you lose more force. The fact that you do what you do, means physically that you cannot go on doing it long; you must have leisure; you must have rest. And if this leisure, if this rest, be not found for you, as a work of mercy to others, our hospitals must break down. Public opinion will do much. You can create, you can enforce it. How? By a quiet persistent assertion of the dignity of your work. You are, each of you, part of a great machine. Assert the dignity of your work by remembering this. Try not to fret against emergencies. Realise that one day you will look back upon things now trivial and irksome as indispensable parts of your training. Shut your eyes to injustice, even to petty spite. It is unworthy of you. Assert the dignity of your work by showing method in it; method in your recreation. You must, or ought to, judge of what recreates you. Restful roaming through green fields, or quiet talk with some dear old person under a shady tree, an absorbing, exciting novel, the glorious swell of sacred music, and is the English stage so hopelessly bad that I may not say a right-some amusing play? Few dare judge you. Your work is a sacred work. You must claim a sacred independence in your recreation; often you know, this is not so. You are wilful in refusing, you are reckless in choosing. I dare not have said this to you this last summer had I seen you in the stifling irritating London heat, but I say it now. You overstrain yourselves out of pure jealousy, you lose a trifle of health and spirits, and you say 'all shall go.' That eternal pile of medicine bottles goads you to desperation, and your soul loathes the smell of a disinfectant. But this is not all, and you know it. There are times when it may be your thoughts are more brilliant, your sayings smarter, your momentary power over a patient more complete, but times too when you cannot bear the smallest rebuke, even an official order which takes no count of your importance. Times when irreverence is most ready on your lips, unbelief most clamorous and loud in your hearts, and God seems a far-off dream. It is then that your danger comes. I will not shrink from saying it; you are grateful even as I say it. You know the use of drugs, their soothing, fascinating power. But here, you say and say truly, knowledge, not ignorance, is your safeguard. I admit it with reserve—But I speak no longer of drugs when I say that sometimes ignorance, not knowledge, is your safeguard, and if you cannot learn this, and if your noblest emotions are exhausted, and if the highest springs of action are, as they sometimes are, weakened by physical overstrain, and if you are thrown off your balance, these are times when in the sacred name of womanhood you will have to fall down upon your knees, and gathering body and soul together, pray 'Lead us not into temptation.' Whatever abuses there may be in hospitals, however faithless individuals, however commonplace and wearisome your work may sometimes be, those outside will always honour the name of nurse. The world will go on reverencing that word. The rules of your institution, the wishes of your chaplain, may or may not cut you off from direct religious intercourse with the sick or dying. Always obey humbly, and be assured that even with this reserve, your secular work will be an incalculable blessing to the souls as well as to the bodies of those committed to your charge."

At the conclusion of the service the visitors strolled about the gardens and grounds enjoying the delicious weather of a perfect autumn day, until they were summoned to an excellent dinner at half-past one. Afterwards the whole party was conducted through the principal rooms by Mrs. Verney, Sir Harry's daughter-in-law, who gave a graphic description of the valuable collection of pictures, &c.

At three o'clock carriages conveyed the probationers for a charming drive, the road lying through woods, until a plateau called Finemoor was reached. Here they alighted and wandered about, and were soon laden with glorious spoils from hedgerows and copse.

Returning to Claydon House, they found an invitingly spread tea under an oak tree on the lawn, and after this popular meal the party of Nightingale Probationers started on their return journey to London, many hearty thanks and good wishes having been expressed to their kind host and his family for the trouble they had taken to make the annual visit a successful and happy holiday.

### In the District.

(By a Novice.)

The different impressions given by things seen for the first time, compared with their appearance when use has made them familiar is perhaps nowhere more remarkable than in matters connected with nursing. I would give much to feel to-day the pleasing sensations of that first morning, when I was taken round by a trained nurse to the district where she ministered to the sick poor in their own homes. I had just arrived at one of those admirable institutions, where ladies are received for two months and put in the way of a little practical work. By the way, why don't people use them more freely? It is invaluable experience in any phase of life, but specially of benefit to persons living in out of the way places, where trained help is so hard to get in emergencies.

The morning after my arrival we started out together. I felt of immense importance in the large cloak and straw bonnet, in which I was protectively clothed by the kind Superintendent of the Home. I gazed longingly at the basketful of treasures, such as rag, soap-plaster, spatula, &c., carried by the nurse, to whom I looked anxiously for all my instruction, for I was profoundly ignorant of the use of those and many other things.

Our first visit was to a most querulous old patient, a Mrs. Smith, who was bedridden and crippled with rheumatism. Nurse washed her all over with infinite care and tenderness, Mrs. Smith keeping up a running comment meanwhile on the hardness of life, the badness of the world, the unkindness and neglect of everyone who had anything to do with herself. I quite longed to point out to her that love and sympathy were being lavished upon her, but probably no amount of explanation could have altered her mental attitude towards the world. Nurse allowed me to do her hair, and the old body blandly remarked, as though the greatest favour in life was mine in being allowed to touch her rather dirty head, "So you like hair-dressing, Miss?" "Nurse, you might just be rubbing my legs a little," and "Nurse, can't you just close the window a bit?" "Nurse, I suppose you couldn't send me a drop of milk," &c.; and as we left the room, quite sure that no more odds and ends could be thought of, she called us back to say, "Nurse, you might just turn me round a little different." From her we proceeded to a similar, yet totally contrary case—the patient also bedridden and helpless from rheumatism; but her gratitude appeared boundless. She realized the luxury of being washed all over and freshened up generally, and every detail done for her comfort was received with such keen appreciation that the most unattractive offices became quite a pleasure.

Our next case was one of varicose veins. The patient, Mrs. Jones, as well as her husband, were quite unique characters. They had never had any children, which made it seem all the more pleasant wit and humour when Mrs. Jones invariably addressed her lord and master as "Papa." They seemed a most devoted couple, and as Nurse cleverly dressed the appalling-looking legs, the woman rambled on about Papa's doings and general superiority. Here the pleasing bumptiousness of the novice found a vent, for a proper nurse would surely not have made a remark so silly as mine. Beyond the nasty smell from legs and ointment, I thought her feet wanted attention, but nothing would induce her to have them washed. She was persuaded it would kill her. I blandly remarked, "Mrs. Jones, I am persuaded that all the mischief could be drawn out of your legs through the feet." (I dared not use the word washing.) "If you would allow us to pour some very hot water over your toes all the mischief from your legs might come out through them." To my amazement she immediately suffered us then, and daily ever after, to wash her toes thoroughly. The treatment ordered by the doctor has improved the condition of her legs, but she is fully convinced that the mischief is departing through the toes.

Mrs. Brown was the next patient on our list, and it was the day for changing her sheets. She was paralysed, and lying like a log. I jumped to the conclusion that her tongue was also paralysed. My astonishment was therefore great when we were just about to remove the soiled sheet, and she said in a deep, loud, deliberate way, "You might take that bug off first, Nurse." I turned away to hide my unseemly mirth, but nurse calmly moved to the offending spot and merely said, "It is only a couple of fleas, Mrs. Brown," and quietly popped the intruders into a basin of water. It would take too long to tell of all the cases we went to that morning, but I certainly gained amusement as well as instruction, and felt that the life of a district nurse was one of great usefulness, calling for immense patience and long suffering. These qualities were certainly to be found in the one I was privileged to work with.

### Appointments.

Fleetwood Cottage Hospital.—Miss Annie McGowan has been appointed Matron of this hospital. She was trained at the Royal Infirmary, Manchester, and has worked for the Manchester Association of Trained Nurses, and takes many good wishes with her for her future success.

Bradford Eye and Ear Hospital.—Miss Margaret Duckitt has been elected from 76 applicants to hold the position of Matron at this hospital. She was trained at the Northern Hospital, Liverpool, where she remained for five years. She was aftewards appointed night superintendent at the Royal Albert Edward Infirmary at Wigan, and then deputy matron at the same institution, with charge of the eye and ear department. Many friends will rejoice to hear of Miss Duckitt's promotion.

### Nursing in South Africa.

(By a Practical Nurse.)

### ROBBEN ISLAND, CAPE TOWN.—THE LUNATIC ASYLUM.

On a brilliant day in August a party of five of us took our places on board the "Tiger," bound for Robben Island. This steamer goes three times a week, and carries supplies, mails, visitors, and fresh patients. On Tuesday the friends of the poor inmates of the asylum and the leper wards are taken across free of charge, and often more than a hundred persons avail themselves of this privilege weekly. On Thursdays and Saturdays other visitors may go on obtaining a pass from the Colonial Office. A charge of 2s. is made for the journey. It was very hot, and we were glad to meet the light breeze as the boat glided across the glassy bay.

Robben Island commands a splendid view of the Blueberg Mountains on the left and Table Mountain and the Lion's Head on the right. It takes the boat three-quarters of an hour to get there. When within two or three hundred yards of the beach we dropped anchor, and a big rowing boat came alongside and took most of the passengers and a good deal of luggage on board, and then rowed as near as possible to the shore. One by one we were then placed on sedan chairs, and borne by means of straps round their shoulders by two convicts, and carried through the tossing breakers. There are always a hundred good-conduct convicts kept for employment on the island, and this landing of passengers is given to them by turns. Immediately in front of us we saw the asylum buildings, fronted by a charming little cottage, with a "stoep" or verandah running all round. We at once made our way to it, and were introduced by one of our party to the Lady Superintendent of the female lunatics. She received us most kindly, and as soon as we had deposited our sunshades, luncheon baskets, &c. (for we have to picnic on the island when visiting it), Miss R. prepared to take us over her part of the asylum. There are between eighty and ninety female lunatics, about equal numbers of white and coloured. They are all bad cases and many of long standing. One old woman is 104, and has been there for the greater part of her life. Unmanageable cases are sent from other parts of the colony; whilst those for whom there seems hope of partial recovery are sent to Falkenberg, an institution two or three miles outside Cape Town.

The coloured patients sleep in wards with seven or eight beds generally, but there are also separate rooms for bad cases, and they have a large dining-room. In fact, they are always kept apart from the white patients, except on Thursdays, when the island band comes to play in the corridor for the patients to dance. This corridor is very long, with an entrance at each end, and little rooms all along both sides. These are for the white patients. There is also a day room with a piano, where some of us played and sang before the dancing began. Everything is open. Not one patient is kept shut up, but all are allowed to wander where they please, within the boundary. The discipline, order, comfort, and exquisite cleanliness of the whole place seemed perfect. The present Matron is admirably fitted for the post, which she has filled with great success for eight years. She has a devoted and excellent staff and a Deputy Matron, who takes her place when she has her well-earned six weeks' holiday.

There are sixteen nurses, including four charge nurses. Their uniform consists of blue serge dresses, white aprons, and blue hoods, instead of caps. As they are in and out of doors all day long, these hoods are much more useful, and in the summer white shady leghorn hats are substituted. The hoods are so pretty and becoming, some lined with red and some with pale blue, as Miss R. said, she could not let a girl with red hair wear a red-lined hood. It was very nice to see the friendly and pleasant terms of the nurses and patients, but it was also very sad to watch the latter and listen to their wild, strange talk, and I was filled with thankfulness that there are those to be found who "serve Him not only with their lips but in their lives, by giving up themselves to His service"; in fact, the whole island seems to testify to a wonderful love and sympathy for poor suffering humanity.

Robben Island is an unique place, such a small area, cut off from the mainland, surrounded by the ever-changing ocean, and containing such a large proportion of suffering humanity. There are three hundred male lepers, and nearly two hundred female!

To return to the male lunatics. They have their wards and enclosure at about two minutes' walk from the other buildings, and just between the two is the apothecary's shop, where all the medicines and ointments are made up for the lepers and the other inhabitants of the island, but it is a curious thing that the lunatic patients scarcely ever have a day's illness, and last year, when there was a great outbreak of typhoid fever, every one of them escaped. It must not be thought that there are only patients and nurses on the island. There are two doctors, a clergyman, a magistrate, a clerk of the works, the convict establishment, the police force, &c., and all the children of these different residents. There are about 1,200 people in all, and of these over 100 are children, and there are two schools for the latter. We were taken over the different wards by the male superintendent, evidently the right person in the right place; we were treated with the utmost courtesy, and the patients evidently experienced kindly firmness. The discipline, order, and cleanliness have much improved since the appointment of the superintendent two years ago.

There are many very bad and difficult cases, but all are allowed perfect freedom, so far as walking about in the courtyards, reading, smoking, &c. One man, quite apart from all others, walking moodily up and down, stabbed to death a former superintendent; and there is also a Chinaman, a very excitable dangerous character; but we had abundant proof in the short time we were there of the general way in which the patients were treated, and nothing I should think could be more satisfactory. There are many separate rooms also long wards with 30 or more beds in. One man, an English merchant, who carried on business at Graham's Town has been over seventy years on this island. He was "crossed in love," and the sorrow affected his brain. Now with long hair and beard, and curious self-made clothes, he looks a veritable Rip Van Winkle; he refuses to sleep anywhere but in a store room where clothes are kept, and has covered the doors and such parts of the walls as have no cupboards, with paintings of curious figures, animals, birds, &c., and is never happier than when demonstrating with a short stick on these productions to an audience.

We went into the kitchen, where half a dozen of the mildest cases work, with two attendants at the head. Great brick fire-places, with steps up to them, and huge caldrons of soup and vegetables; one grey-headed old man washing down the zinc-covered table, had been an inmate for forty years; another was milking the cows. Work is apportioned as each is able and fit for it. There are about seventy-nine white and coloured male patients. From the little railed-in garden, with neat shell paths, we were each asked to take a button-hole of the sweet English wallflower or mignonette, or the delicate white freezia, with its exquisite scent, or the Cape forget-me-not, a much deeper intenser blue than our little sky-like flower. Then we had to say good-bye, for the leper establishment had to be visited before lunch.

### Everybody's Opinion.

[Correspondence on all subjects is invited, but we cannot in any way be responsible for the opinions expressed by our correspondents. No communications can be entertained if the name and address of the correspondent is not given, or unless one side of the paper only be written on.]

### NURSING DEFECTS IN COTTAGE HOSPITALS.

"A Senior Practitioner" writes: The discussion as to the nursing arrangements in Watford Cottage Hospital may be considered closed, but a more important question remains behind it, namely, that a nearly identical system is followed in many small hospitals, with the result that the health of the nurse is impaired and the nursing rendered inefficient. The managers of small hospitals, which generally have from six to twelve beds, seem to think that because the patients are few, one trained nurse with, and often without, the aid of a probationer is sufficient. They forget or ignore the fact that one or two patients often require as much care as twenty; that to neglect even one patient is a serious dereliction of duty, and that it is not the actual work alone, but the constant strain on a conscientious nurse that tells. In many of these hospitals the nurse either sleeps in the ward with the patients, or in an adjoining room into which a bell rings. In either case she is liable to be roused from her sleep to attend to the wants of the patients, and if one of these be seriously ill, or unreasonable, it becomes a grave matter for her; for in addition to the risk of being chilled, such disturbed rest is hardly to be preferred to sitting up all night. I have known a nurse for several nights in succession to have no sleep, except such snatches as could be obtained while sitting up, preferring this to the alternative of getting out of bed. Frequently the result is weariness and exhaustion, and as a necessary consequence her duties must be inefficiently performed and the patients badly attended to; and the nurse who is thus on duty day and night is hardly to be blamed for this. The fault rests entirely on the managers. But even if neither a serious nor a troublesome patient is under her care she does not get refreshing sleep—she feels that the cases are still under her charge. She becomes first watchful and then wakeful. I have known a conscientious nurse get up uncalled more than once in the night to see if all be right; and even if she does not get up she will be on the watch; and so in time health gives way, and another is added to the list of invalids made such by trying to relieve others. All this is wrong. No hospital, however small, should be without a night nurse; the number of patients received makes no difference—one patient demands her presence as much as twenty; and if the managers of these cottage hospitals cannot afford to have a competent night nurse, it would be far better for them to close the hospital altogether. To get, as is sometimes done, some old woman to come in occasionally and sit up at night is no remedy. A competent nurse is more needed by night than by day, and many a patient whose life might have been saved has died from the incompetence of the so-called nurse left in charge at night. I trust that the lesson to be learned from the Watford Hospital will not be without its use, and that of Miss Hillyer's broken health will at least save others from a similar fate.

### A TRAINED NURSE IN DIFFICULTIES.

"Mr. R. Gofton Salmond, Hon. Secretary Trained Nurses' Annuity Fund," writes: May I solicit your help in finding a home for a trained nurse aged 61, who has been for several years incapacitated by incurable tumour, and for which no relief can be obtained by operation? This nurse, having recently lost her husband, is now without a home, and I am anxious to find her one with some one who will take an interest in her and attend upon her with kindness and give her every comfort, for which due payment will be made. Perhaps amongst your readers there are many who would care to provide a home for this their suffering sister. One of Evangelical principles preferred.

### For Reading to the Sick.

### BELLS.

How sweetly the sound of a distant peal of bells falls on the ear. When the air is calm and still, and the setting sun is sinking to his rest, they call us to prayer, and whisper words of comfort and peace to the soul; while on a winter's night, as they resound through the crisp, frosty sky in joyous cadence, the heart echoes the glad tones with cheerful thankfulness as they recall to our minds the words of the beautiful hymn—

"Whene'er the sweet Church bell
Peals over hill and dell
May Jesus Christ be praised;
O, hark to what it sings,
As joyously it rings,
May Jesus Christ be praised."

Yes,

"Far, far away, like bells at evening pealing,
The voice of Jesus sounds o'er land and sea,
And laden souls, by thousands meekly stealing,
Kind Shepherd, turn their weary steps to Thee."

Bells, again, have another duty to perform. Amid the stormy wind and tempest the lighthouse bell warns the mariner to shun the treacherous rocks which are lying in his path. Bells, too, enter into all our joys and sorrows; they ring out merrily for a wedding and solemnly toll the knell of a departed friend.

It would be well if we could always have a peal of grateful thoughts in our hearts, but I suppose that is very difficult—you who are sick and suffering will say "impossible." Dear friends, let us try to have a chime ready to hand; if we "set" our bells, as the bell-ringers call it, we can have sweet music at any moment, and as "A cheerful heart doeth good like a medicine," we shall require fewer bitter draughts from either our earthly or our heavenly Physician to correct our ailments of body or of soul. We will not dwell continually on dismal subjects, or be ever tolling the knell of departed joys. Neither will we look on the dark side of the possible future, nor pick holes in our friends and neighbours. These are not the bells which cheer our hearts, or even warn us of difficulties in our path. We may never muster up a full peal of eight or twelve bells, but a great deal may be done with the three principal ones of Faith, Hope, and Love. Let us ring the changes on those, and we shall lack nothing in our harmony.

There is yet another use to which bells are put, namely, preparation for worship. We learn from the book of Exodus that God commanded Moses to sew bells of pure gold on the hem of the High Priest's robe, that as he moved through the sanctuary the worshippers should prepare their hearts to go into the presence of the Almighty whom they had assembled to adore. When we are about to pray let us cast out of our minds all foolish wandering thoughts which would alloy the pure gold of devotion, and be careful to have reverent souls and bodies which will help to make our prayers acceptable to Our Father which is in Heaven, and make our hearts and minds ring out with the refrain, "May Jesus Christ be praised."

### Minor Appointments.

Fountain Fever Hospital.—Miss Mary Gamion has been made Charge Nurse at this hospital. She was trained at the London Hospital, and afterwards worked for nine years in connection with the Holles Street, Dublin, Nurse Training Institute, and for the last year has been Charge Nurse at Park Hill Hospital, Liverpool. Miss Gamion's testimonials are excellent.

The recent changes in the medical and surgical staff at St. Thomas's Hospital have brought about alterations in the nursing staff. Miss Howes has been appointed Night Superintendent, Miss Haig-Brown transferred from Elizabeth to Alexandra, Miss Gaston to Elizabeth, and Miss Young to Edward ward.

### Holiday Expeditions.

(By a District Nurse.)

### I.—MONT BLANC.

After a few pleasant days at Chamonix, during which we made several walking expeditions and did some easy climbing, Nurse W—— and I resolved to make a more exciting attempt. We therefore engaged two guides to accompany us to the Grands Mulets, nearly 7,000 feet above Chamonix, and more than half-way up Mont Blanc. So one fine morning in September we left Chamonix at nine, and walked by the zig-zag path through the woods up to Pierre Pointue, where the guides insisted on an hour's rest to take their mid-day meal, while we sat on the rocks and enjoyed the light luncheon we had carried with us. The ascent from Pierre Pointue continues for nearly an hour by a narrow winding path along the side of the moraine by the Glacier des Bossons. From various points on the route glimpses of the great glacier, with its enormous glistening pyramids of ice, were obtained, and beautiful peeps of the valley—the village of Chamonix far below us, the clustered habitations showing like toy houses, and the River Arve reduced to a mere silver thread. After some scrambling over loose boulders, we gained the Pierre à l'Echelle, and a few minutes later we were on the ice. The first part of the glacier journey was quite easy, but by degrees the path became more difficult, and very soon our leading guide announced that we must now be "roped." The walk now began to be truly exciting. We passed over chasms of indefinite depth, skirting the edges of grand glacial precipices, and ever-toiling gradually upwards. Where the crevices were practicable, we jumped across, and where they widened to an impassable distance, ladders were used to bridge the gulf. In some places the guide had to cut steps in the ice walls with his axe, and we followed cautiously, not looking beyond our next step, on the firmness of which depended our safety.

We reached Grands Mulets about four p.m., and received a warm welcome at the hut, and some tea was most acceptable, for we were beginning to realise that we were 10,000 feet above the sea, and felt decidedly chilly. We watched the sun go down from the little platform, and a grand, though dreary, scene it was, till the cold fairly drove us in, and we were glad to sit as near as possible to the little kitchen stove, where the guides' supper was being prepared. As soon as this was served, our dinner was got ready—a wonderful meal, considering that everything had to be carried up from the valley and across that great glacier. Six courses! well cooked and well served, with snowy table linen, and dessert and wine. Dinner over, there was nothing to do but go to bed, and soon after seven we turned in, and lay listening to the wind howling round the hut, and wondering what the morrow would be like, for by this time, finding myself within 5,000 feet of the summit, I had decided that if the weather was at all favourable I would try and get to the top of Mont Blanc. We did not sleep much: it was all too strange, and the noise of the wind was not soothing. At one a.m. I rose to consult the guides as to the weather. They would not give an opinion. "Not very good," "Not very bad," "A good deal of wind," and many shrugs of the shoulders.

So finding that the decision lay entirely in my own hands, I told them to pack the necessary provisions, and at two a.m. we started, a silent procession of three, all roped together, and the leading guide carrying a lantern. My friend had decided to await us at Grands Mulets, and she scarcely recognised me when I went in to say "Au revoir!" for, in addition to my ordinary climbing attire, my face and head were muffled up in a woollen shawl under my hat; I wore thick felt gaiters coming up to the knees; woollen mittens over my gloves, with blue spectacles to put on when the sun rose. There was no moon, but the stars shone brightly, and it was a clear, cold morning. We had several delays, caused by our lantern being extinguished by the wind, and it was no easy matter to re-light it. However, the dawn appeared at last, and the lantern was left in the snow, to be picked up on our return journey. We reached the Grand Plateau (12,900 feet) as the day was breaking and as the sun rose, and peak after peak appeared, bathed in rosy light. It was like fairyland, and the scene gradually warmed and broadened cheerfully. The cold was now intense; the guides had icicles hanging from their moustaches, and the woollen shawl round my face was hard and stiff with ice. At the farther extremity of the plateau we attained the shelter of the Rochers Rouges, and rested for about twenty minutes in the cabane there to take some refreshments. Carefully and slowly we then continued our route, step-cutting being again necessary here. The wind was very strong and blew the snow about in clouds, stinging our faces like so many needles, and I began to wonder whether I could go any farther, for now and then the gusts nearly carried me off my feet. However, the guides bade me take courage, and said we were only three hours from the summit; so on we went. The "calotte" of the mountain is difficult to climb, being covered with loose frozen snow, and I sank in up to the knees at every step.

At last we arrived, tired, but triumphant, on the top after eight hours' steady walking and climbing, and it was grand to feel oneself standing on the very highest spot in Europe (15,731 feet). Right glad I was to sit down by the stove in the observatory which has just been completed by Professor Janssen, and gratefully did I accept some "bouillon" which he very kindly ordered his men to give me. I was invited to place my feet in the oven, and a pie which was baking there was removed in order that I might do so! I felt very drowsy, and should have been glad to have a nap, but my guides would not hear of such a thing, and after about ten minutes' rest they insisted on starting. The descent is comparatively easy, though there are places where great caution is necessary, and a false step might lead to serious consequences. And now we were glad of our blue spectacles, for the sun shining on the snow made a blinding glare. We picked up our lantern as we came down, and reached Grands Mulets at half-past four p.m., where I found Nurse W—— anxiously awaiting our return, for, on account of the strong wind, we had been a good deal longer than we anticipated. I begged for half an hour's rest and "a cup of tea," and at five we again started to cross the glacier on our homeward route. Our guides thought we should have time to get off the ice before dark, and we walked as quickly as possible in order to do so, but after a glorious sunset it became dark very quickly, and after a short time our leading guide stopped suddenly and looked all round in a hesitating way, and we realised that we were "lost on the glacier." We had absolutely nothing to show us the way, and time after time we had to turn back and try new routes, jumping across crevasses that we should have hesitated to attempt in broad daylight. It was nervous work, but I felt confident that the guides would get us off somehow, and at last their efforts were crowned with success, and we were once more on the moraine and "unroped," and after a short time we found ourselves back at Pierre Pointue, where we insisted on the guides borrowing a lantern to light up our homeward path.

At half-past ten p.m. we reached our hotel, where we had a great reception, and were presented with large bouquets; and truly thankful were we to have some supper and get to bed. I had been just twenty hours on foot—not a bad day's work, even for a district nurse!

### Wants and Workers

Regular Employment during the winter could be secured for a good needlewoman and cutter-out, by application to Miss Simeon, 4, Granville Terrace, Ramsgate.

### The Book World for Women and Nurses.

[We invite Correspondence, Criticism, Enquiries, and Notes on Books likely to interest Women and Nurses. Address, Editor, The Hospital (Nurses' Book World), 428, Strand, W.C.]

General Rules for Guidance of Maternity Nurses. (Ladies' Sanitary Association, 22, Berners Street. Price one halfpenny.)

This is an excellent little guide, full of common sense, and giving thoroughly safe hints for the use of ordinary nurses. We can most cordially recommend it to the maternity nurse, whom it is specially designed to assist.

The Thumb Prayer-book (Oxford University Press, Amen Corner.)

The University Press have just brought out the most delightful and convenient edition of the Prayer-book. Although the little volume weighs but three-qnarters of an ounce, the printing is so good as to be equally legible as in larger edtiions. It is printed on Oxford India paper, and well and plainlybound. We cannot imagine a more useful present for Christmas than this little volume, which encroaches on so small amount of space, and is far more conveniently handled than the longer shaped books we are accustomed to associate with the name of Thumb Prayer-book.

"Facts About Disinfectants." By H. Helbing and Dr. F. W. Passmore. (Published at 63, Queen Victoria Street, E.C. Price 6d.)

This little pamphlet gives some useful facts about disinfectants, and the whole leads up artistically to the main object in view—the great advantage of coal-tar preparations as disinfectants. We cannot, however, agree with the authors in thinking that legislation is needed with regard to disinfectants, and that we want another Act, similar to the Food and Drugs Act, to protect disinfectants and other similar substances. We think there is grave risk in over-legislation. We have plenty of reliable disinfectants in the market, and the public cannot do better than trust to these in cases of infectious disease. The public, however, never will do this; it will try new things, and no legislation will prevent it, nothing but bitter experience oft repeated. Much can be done by medical practitioners and nurses in cases of infectious disease, and we believe that if these would give careful and precise directions about disinfectants, and would distinctly say what disinfectants are to be used, that there would be no call for legislation, or for the lament of the writers of this pamphlet over the gullibility of the public in the matter of substances which are disinfectants only in name. We think it a pity that the authors have not made any distinction between disinfectants and antiseptics, by which omission they obscure their meaning. Then in the list of "properties which a good disinfectant must possess," we find classed together "activity against spores," and "innocuousness to human life," properties which are absolutely incompatible. There is no doubt that the various coal-tar preparations have proved most useful as antiseptics, and will therefore hold their own, but in cases of the most serious infectious diseases we should be sorry to have to trust only to disinfectants of which the chief merit is their "innocuousness to human life."

**THE BOOK WORLD FOR WOMEN AND NURSES**—*continued.*

### A NEW JOURNAL.

"The Woman at Home," the last new ladies' paper, edited by Annie Swan, is rather vapid in its first number. However, as it appears to wish to cater for the ladies of the lower middle classes, who are probably unable to afford the more dashing ladies' weeklies, it may be "filling a long-felt want." A paper "written round" ancient photos of "our Princess" is wisely inserted, as the love and interest of the nation is such that even an article that tells nothing new is welcomed and bought for her sake. Madame Sarah Grand's name being the next best advertisement, she has been induced to furnish a tale which was probably written while she still wore pinafores. The illustrations make it appear that she breakfasted while in China in a very *decolleté* dinner gown on a large bowl of flowers, half an antimacassar, and a teapot with no spout. Many pages are devoted to a reprint of the fashionable weddings which took place in June. These are very stale reading in October, but perhaps may help to decide the winter fashions of the back shop. A photograph of Olga Beatty-Kingston suggests a child of ten years old at the outside. Either Olga has inserted a very old photo, or Olga is a terrible little prig, and writes about M. Pasteur in a ridiculous fashion for so young a child. Barones von Zedlitz was also a Miss Beatty-Kingston, we believe. She contributes an adoring article on Madame Patti and a very ugly photo of Craig y Nos Castle. Madame Patti's self-love is naively illustrated by her answers in a confession album. "Which is your favourite story?" Answer: "My own." "The Experiences of a Lady Doctor" is so profusely illustrated that it would seem the authoress wishes to save the reader all fatigue of the imagination. The story has a wholesome moral tone, although the style is not powerful nor does the series promise any great originality. Maarten Maarten's little story is touching. It tells of a painter who has struggled all his life in vain to sell his pictures, while his devoted wife, by sheer hard work, keeps him and herself body and soul together on the slenderest pittance. After twenty-five years she dies worn out, and the broken-hearted husband spends the days in gazing at portrait painted of her in her youth by a friend who became a greater and more successful painter than himself. His landlady comes to him for money wherewith to order the funeral; but not a groschen is to be found. She persuades a dealer to come and look at the pictures in the hope that he may buy some. The dealer comes, speaks kindly, but shakes his head over the work. Then catches sight of the portrait. "Ah! a genuine Gleismann!" He offers to purchase it, but will buy nothing else. To save his adored wife from a pauper's funeral the poor painter parts with the only treasure which remained to make life sweet to him. "Dress and Fashion" is illustrated very appropriately from models of Messrs. Evans in Oxford Street. The cookery receipts include chicken mayonnaise and boiled ham. The suggestions for furniture may be improvements on the correspondent's own tastes, but we shudder to think so. Nor do we think that children would be so interested as the writer imagines by the statistics of the food bill at the Zoo.

